# Genome-Wide Association Study of Serum Minerals Levels in Children of Different Ethnic Background

**DOI:** 10.1371/journal.pone.0123499

**Published:** 2015-04-17

**Authors:** Xiao Chang, Jin Li, Yiran Guo, Zhi Wei, Frank D. Mentch, Cuiping Hou, Yan Zhao, Haijun Qiu, Cecilia Kim, Patrick M. A. Sleiman, Hakon Hakonarson

**Affiliations:** 1 The Center for Applied Genomics, Children’s Hospital of Philadelphia, Philadelphia, Pennsylvania, United States of America; 2 Department of Computer Science, New Jersey Institute of Technology, Newark, New Jersey, United States of America; 3 Department of Pediatrics, The Perelman School of Medicine, University of Pennsylvania, Philadelphia, Pennsylvania, United States of America; 4 Division of Human Genetics, Children’s Hospital of Philadelphia, Philadelphia, Pennsylvania, United States of America; Emory University, UNITED STATES

## Abstract

Calcium, magnesium, potassium, sodium, chloride and phosphorus are the major dietary minerals involved in various biological functions and are commonly measured in the blood serum. Sufficient mineral intake is especially important for children due to their rapid growth. Currently, the genetic mechanisms influencing serum mineral levels are poorly understood, especially for children. We carried out a genome-wide association (GWA) study on 5,602 European-American children and 4,706 African-American children who had mineral measures available in their electronic medical records (EMR). While no locus met the criteria for genome-wide significant association, our results demonstrated a nominal association of total serum calcium levels with a missense variant in the calcium –sensing receptor (*CASR*) gene on 3q13 (rs1801725, *P* = 1.96 × 10^-3^) in the African-American pediatric cohort, a locus previously reported in Caucasians. We also confirmed the association result in our pediatric European-American cohort (*P* = 1.38 × 10^-4^). We further replicated two other loci associated with serum calcium levels in the European-American cohort (rs780094, *GCKR*, *P* = 4.26 × 10^-3^; rs10491003, *GATA3*, *P* = 0.02). In addition, we replicated a previously reported locus on 1q21, demonstrating association of serum magnesium levels with *MUC1* (rs4072037, *P* = 2.04 × 10^-6^). Moreover, in an extended gene-based association analysis we uncovered evidence for association of calcium levels with the previously reported gene locus *DGKD* in both European-American children and African-American children. Taken together, our results support a role for *CASR* and *DGKD* mediated calcium regulation in both African-American and European-American children, and corroborate the association of calcium levels with *GCKR* and *GATA3*, and the association of magnesium levels with *MUC1* in the European-American children.

## Introduction

Calcium, magnesium, potassium and sodium cations as well as chloride and phosphorus anions are major dietary minerals which cannot be manufactured by the human body. They play important roles in various chemical and biological functions and are essential to sustain life and maintain optimal health. As a result, these minerals are commonly measured in the blood serum to monitor a range of health conditions. Sufficient mineral intake is especially important for children and adolescents due to their rapid growth.

Consistent with the high heritability estimates of serum mineral concentrations (calcium, 21–45%; magnesium, 15–39%; phosphorus, 53–62%) [1–4], previous genome-wide association studies (GWAS) have identified common variants at multiple loci (*CASR*, *CYP24A1*, *DGKD*, *GCKR*, *CARS*, *DGKH*, *GATA3*, *VKORC1L1*), influencing serum calcium levels [5–7]. Common variants at six other gene loci (*MUC1*, *ATP2B1*, *DCDC5*, *TRPM6*, *SHROOM3*, and *MDS1*) were also shown to be associated with serum magnesium levels [8]. However, no gene locus has been reported in other dietary mineral quantitative traits and no pediatric or African-American cohort has been investigated so far.

In this study, we performed a genome-wide association study to explore the contribution of potential common genetic variations to serum concentrations of the six major dietary minerals in children of both European Caucasian and African-American ancestry. A total of 10,308 children (5,602 European-Americans and 4,706 African-Americans) were included in the analysis. All samples were genotyped on the Illumina HumanHap550 or Quad610 arrays, having ~530,000 SNPs in common. The average serum levels of the six minerals were tested for associations by linear regression as implemented in PLINK [9]. The SNP associations were further used for a gene-based association analysis and the significant genes were mapped to protein-protein interaction (PPI) network, as previously described [10].

## Materials and Methods

### Ethics statement

This study was approved by the Research Ethics Board at the Children's Hospital of Philadelphia (CHOP) and written informed consent was obtained from all research subjects and/or their parents by nursing and medical assistant staff under the direction of CHOP clinicians.

### Sample collection

10,308 subjects were recruited from The Children’s Hospital of Philadelphia (CHOP) Health Care Network. To identify genetically inferred European-American and African-American children, we first plotted the first two principal component vectors generated by EIGENSTRAT and then used k-means (k = 5) clustering to partition the subjects into subgroups [11] (**S1 and S2 Figs**). Details of the European-American and African-American cohorts used in this study are summarized in **S1 Table**. The serum levels of calcium and magnesium displayed normal distribution in both European-American and African-American cohorts (**S3 Fig**). Subjects with missing data or measurement beyond 3SD of the mean were removed from the study. For the calcium levels, 5,261 European-American children and 3,817 African-American children were included after quality controlling the data. For the magnesium levels, 2,317 European-American children and 1,283 African-American children were included after quality control.

### Statistical analysis

All subjects were genotyped by Illumina 550K SNP array and Human610-Quad version 1 array following the Illumina standard protocols as previously described [12]. Samples with genotype call rate above 95% were included in the study. SNPs with genotype missing rate >5%, minor allele frequency <1%, and Hardy-Weinberg equilibrium P value <0.00001 were included. 495,328 and 507,950 markers were included for the association test of serum calcium levels in the European-American and African-American children respectively. 495,763 and 509,150 markers were included for the association test of serum magnesium levels in European-American and African-American children respectively. The analysis was implemented by PLINK [9].

The association analyses were carried out in PLINK using linear regression models. The additive model was used for the association test. The meta-analysis was performed using fixed effects models in PLINK. (**S4 and S5 Figs**). We used age, gender and the first ten PCs calculated by EIGENSTRAT as covariates and the genomic inflation factors were 1 for all the analysis (**S6 and S7 Figs**).

The gene-level significance was calculated by VEGAS based on the p values of the SNPs positioned within 50kb upstream and 50kb downstream of the gene boundaries [13]. VEGAS can perform Monte-Carlo simulations from the multivariate normal distribution based on the LD pattern from the reference populations and assign an estimated p value to each gene. The meta-analysis of the gene-based p values was performed by Fisher’s method. The significant genes (p value <0.05) were considered for further PPI network analysis.

### Protein-Protein interaction network construction

The PPI Network was constructed based on the database iRefindex, which collected the protein interactions from a number of primary interaction databases [14]. In order to obtain a high-confidence network, we only kept the human-human protein interactions reported by at least two Pubmed articles (For example, if a viral protein interacts with a human protein, this is counted as human interaction by iRefindex; we only consider the interactions from both human proteins). At last, a reference network of 9,090 nodes (proteins) and 25,864 edges (interactions) were constructed.

We next mapped the nominally significant genes (*P* < 0.05) calculated by VEGAS to the reference network. The subnetwork was constructed by the significant genes and the interactions among them. The subnetwork contains a few connected components and many singletons. We then extracted the largest connected component (LCC) of the subnetwork. The same list of genes will generate exactly the same LCC. Normally, the LCC is much bigger than other connected components. To investigate the biological significance of the genes in the LCC, we carried out a gene function enrichment analysis against the KEGG and BIOCARTA database using DAVID [15].

## Results

We carried out a GWA study on our pediatric cohort including 5,602 children of European-American ancestry and 4,706 children of African-American ancestry, where we investigated the associations between genotyping markers and six dietary mineral traits, including calcium, magnesium, potassium and sodium cations, and chloride and phosphorus anions. Although no genome-wide-significant signal was detected due to the lack of power, we confirmed a few previously identified loci (**S2 Table**).

In the African-American children, we report the association of total serum calcium levels with a missense variant in the calcium-sensing receptor (*CASR*) gene on 3q13 (rs1801725, *P* = 1.96 × 10^–3^, *P–adj* = 7.84 × 10^–3^, regression coefficient Beta = 0.09, minor allele T, **Table 1**). The SNP was previously reported to associate with serum calcium levels in both European Caucasian and Indian-Asian populations, but no previous studies have been conducted in African-American population or pediatric populations [5–7]. The association between the rs1801725 variant and the total serum calcium level was also confirmed in our European-American pediatric cohort (rs1801725, *P* = 1.38 × 10^–4^, *P–adj* = 5.52 × 10^–4^, Beta = 0.05, minor allele T, **Table 1**). Thus, our results extend for the first time the previous observations of *CASR* mediated calcium regulation in adults of European ancestry to children of both African-American and European-American origin. We further replicated two other loci associated with serum calcium levels in the European-American cohort (rs780094, *GCKR*, *P* = 4.26 × 10^–3^, *P–adj* = 0.02, Beta = 0.03, minor allele A; rs10491003, *GATA3*, *P* = 0.02, *P–adj* = 0.08, Beta = 0.04, minor allele A; Table 1). In addition, our results confirmed another previously reported association of serum magnesium levels with the variant rs4072037 (*P* = 2.04 × 10^–6^, *P–adj* = 8.16 × 10^–6^, Beta = -0.03, minor allele G, **Table 1**) located in *MUC1* on 1q21. In contrast, the rs4072037 variant was not associated with the magnesium level trait in the African-American children (**Table 1**). All the directions of the associations confirmed in our study were consistent with those reported in previous studies. For the other dietary minerals (potassium, sodium, chloride and phosphorus), no significant locus was found to be associated in our dataset.

**Table 1:** Associations between serum calcium/magnesium levels and the replicated SNPs surpassing genome-wide significance in previous studies.

**Trait**		Ca	Ca	Ca	Mg
**Gene**		*CASR*	*GCKR*	*GATA3*	*MUC1*
**Region**		3q21	2p23.3	10p14	1q22
**SNP**		rs1801725	rs780094	rs10491003	rs4072037
**A1/A2**		T/G	A/G	A/G	G/A
**European American**	**Beta**	0.050	0.028	0.039	-0.028
	**SE**	0.013	9.7E-03	0.016	5.8E-03
	**P**	1.4E-04	4.3E-03	0.018	2.0E-06
	**P-adj**	5.5E-04	1.7E-02	0.072	8.2E-06
**African American**	**Beta**	0.085	-0.023	6.3E-03	-0.012
	**SE**	0.028	0.014	0.016	8.4E-03
	**P**	2.0E-03	0.10	0.69	0.16
	**P-adj**	7.8E-03	0.40	1.0	0.64
**Combined**	**Beta**	0.057	0.011	0.022	-0.023
	**P**	1.8E-06	0.16	0.052	2.4E-06
	**P-adj**	7.1E-06	0.64	0.21	9.5E-06

A1/A2: minor allele/major allele (The minor allele/major allele is the same in European-American children and African-American children)

Beta: Regression coefficient (unit, mg/dl)

SE: standard error of Beta

P-adj: P-value adjusted by Bonferroni correction (based on four examined SNPs)

We next sought to infer the gene-level significance from our SNP associations and utilize the prior knowledge from protein-protein interaction networks [10, 16]. To that end, we used a versatile Gene-Based Test approach (VEGAS) to convert the individual SNPs into gene-wise p values [13]. The significant genes (gene-wise p value below 0.05) were selected for downstream analysis (**S3 Table**). For the gene-based test, we found that the previously identified genome-wide significant locus on 2q37.1 (*DGKD*) was significant in both the European-American and African-American children (**S3 Table**).

The significant genes from both European-American and African-American cohorts were further mapped to the reference PPI network. For the calcium trait, we identified a subnetwork containing 815 nodes and 290 edges, and we found that genes of the largest connected component (LCC, 172 nodes, 196 edges, **S8 Fig**) in the subnetwork were enriched in ‘TGF-beta signaling pathway’ (*P* = 8.42 × 10^–4^, **S4 Table**). For the magnesium trait, we identified a subnetwork containing 782 nodes and 270 edges, and we found that the most enriched pathway of the largest connected component (LCC, 143 nodes, 164 edges, **S9 Fig**) was ‘Cell cycle’ (*P* = 0.002, **S4 Table**). However, none of the p values of the enriched pathways was less than 0.05 after multiple testing (**S4 Table**).

## Discussion

The product of the *CASR* gene binds to calcium molecules, which can monitor and regulate the amount of calcium in the blood [17]. Previous studies show that mutations in the *CASR* gene can cause Mendelian disorders, wherein loss-of-function mutations lead to familial hypocalciuric hypercalcemia (FHH), while gain-of-function mutations can give rise to autosomal-dominant hypocalcemia or type 5 of Bartter syndrome [7]. Two previous GWA studies also found common variants in *CASR* are associated with serum calcium levels in Caucasians and Indian Asians [5, 7]. Here, we report associations between common variants in the gene *CASR* and serum calcium level not only in European-American children but also in African-American children, further demonstrating that the *CASR* gene plays an important role in calcium regulation across the age span and major ethnicities.

In a previous study [5], the authors identified a strong association between SNP rs1801725 in gene *CASR* with serum calcium levels in the CoLaus European cohort (*P* = 7.56 × 10^–12^, 5,404 subjects) and the Indian Asian cohort (*P* = 1.07 × 10^–21^, 3,947 subjects). In comparison, the associations identified in our cohorts are less robust (the European-American cohort, *P* = 1.38 × 10^–4^, 5,261 subjects; the African-American cohort, *P* = 1.96 × 10^–3^, 3,817 subjects). Since the sample sizes are similar, the variability in developing children of different ages could result in a lack of power to identify genome-wide significance. For example, the age covariance is very significant and much more significant than gender and the PCs indicating a strong association between age and serum mineral levels in our study. It is well known that the serum mineral levels are correlated with the age of children due to their rapid growth, and become stabilized in adult. In addition, environment factors such as diet and physical activity could have a greater influence on the mineral levels in children than adults.

*DGKD*, *GCKR* and *GATA3* were identified in a recent meta-analysis of GWAS in European ancestry individuals [6]. However, the mechanism by which *DGKD*, *GCKR* and *GATA3* influence the serum calcium level is still unclear [6]. Here, we replicated *GCKR* and *GATA3* gene loci in European-American children, but not in African-American children, suggesting these loci may be specific to European ancestries. Although not replicated at the SNP level, *DGKD* was significant in both the European-American and African-American children in the gene-level tests, indicating *DGKD* may also influence the serum level of calcium in the African-American children.

*MUC1* is a member of the mucin family, which encodes a membrane bound, glycosylated phosphoprotein named mucin 1. A previous GWA study has reported common variants at this genomic region that are associated with serum magnesium levels in individuals of European descent [8]. We confirmed this result in an independent pediatric European-American cohort. However, no significant association was identified in our pediatric African-American cohort, suggesting the *MUC1* locus may be specific to European ancestries.

Since relatively few mineral-related loci have been discovered by the classical GWA approach, we employed a network-based pathway analysis method to determine if functionally related genes may collectively contribute to certain serum mineral traits [10, 12]. We uncovered a group of genes of functional interest enriched in the ‘TGF-beta signaling pathway’ that are associated with the calcium concentration. The ‘Cell cycle’ function was associated with both calcium and magnesium levels. Although the enriched functions were not significant in our study following multiple testing, they could be confirmed in future GWAS with a larger sample size.

In conclusion, our results support a role for *CASR* mediated calcium regulation in both African-American and European-American children, and corroborate association of calcium levels with *GCKR* and *GATA3* and association of magnesium levels with *MUC1* in European-American children. Moreover, in an extended gene-based analysis, we uncovered evidence for association of calcium levels with *DGKD* in both European-American and African-American children. We anticipate that future pediatric studies with greater sample sizes will be able to uncover additional associations that will further delineate the genetic underpinnings of these traits.

## Supporting Information

S1 Fig Population stratification of the serum calcium association study. The first two principal component vectors generated by EIGENSTRAT were plotted. European-American cohort were colored in blue. African-American cohort were colored in red. In general, black, brown and green dots denote Asian, Hispanic and Multiracial Americans.
(PDF)

S2 Fig Population stratification of the serum magnesium association study. The first two principal component vectors generated by EIGENSTRAT were plotted. European-American cohort were colored in blue. African-American cohort were colored in red. In general, black, brown and green dots denote Asian, Hispanic and Multiracial Americans.
(PDF)

S3 Fig Distributions of serum calcium and magnesium levels in European-American cohort and African-American cohort. (PDF)

S4 Fig Manhattan plots of serum calcium level GWAS. (PDF)

S5 Fig Manhattan plots of serum magnesium level GWAS. (PDF)

S6 Fig Quantile-Quantile plot of the expected and observed P-values for the serum calcium level GWAS. (PDF)

S7 Fig Quantile-Quantile plot of the expected and observed P-values for the serum magnesium level GWAS. (PDF)

S8 Fig PPI network generated by significant genes identified with VEGAS for calcium trait. (PDF)

S9 Fig PPI network generated by significant genes identified with VEGAS for magnesium trait. (PDF)

S1 Table Details of European-American and African-American cohorts used in the study. (XLSX)

S2 Table Details of top SNPs (P<10^–4^) in this study. (XLSX)

S3 Table Results of gene-based analysis from VEGAS. (XLSX)

S4 Table Enriched pathways of the LCCs. (XLSX)
