# Supplementary figures and images for: Genome-Wide Association Study of Serum Minerals Levels in Children of Different Ethnic Background
Source: PLoS One. 2015 Apr 17;10(4):e0123499. doi: 10.1371/journal.pone.0123499 (PMC4401557; doi:10.1371/journal.pone.0123499)

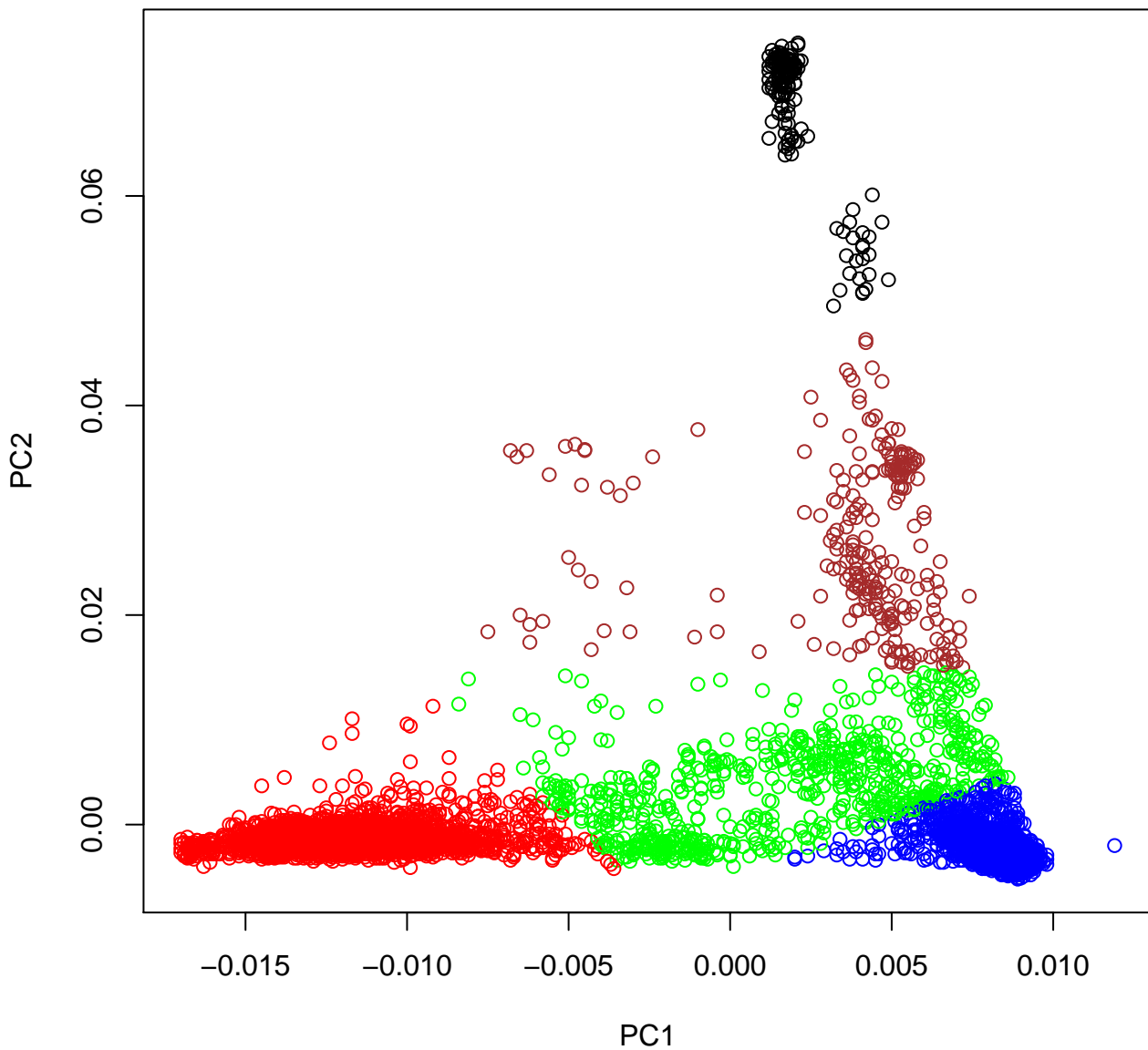

Supplement: S1 Fig — The first two principal component vectors generated by EIGENSTRAT were plotted. European-American cohort were colored in blue. African-American cohort were colored in red. In general, black, brown and green dots denote Asian, Hispanic and Multiracial Americans. (PDF) [file pone.0123499.s001.pdf]

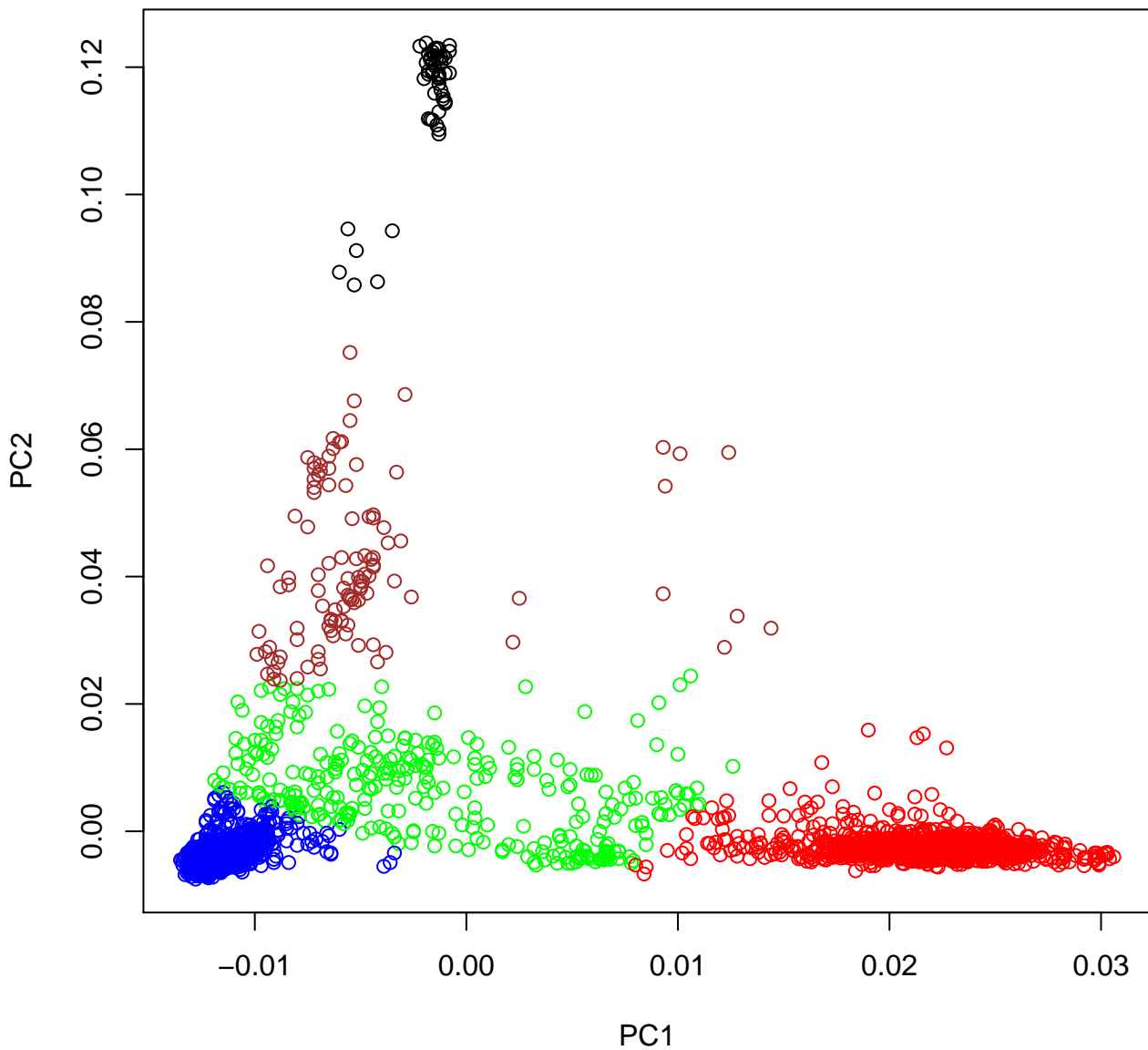

Supplement: S2 Fig — The first two principal component vectors generated by EIGENSTRAT were plotted. European-American cohort were colored in blue. African-American cohort were colored in red. In general, black, brown and green dots denote Asian, Hispanic and Multiracial Americans. (PDF) [file pone.0123499.s002.pdf]

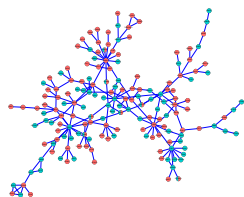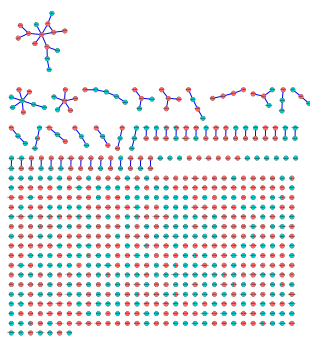

Supplement: S8 Fig — (PDF) [file pone.0123499.s008.pdf]

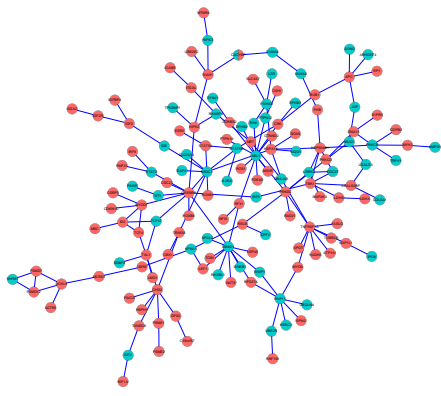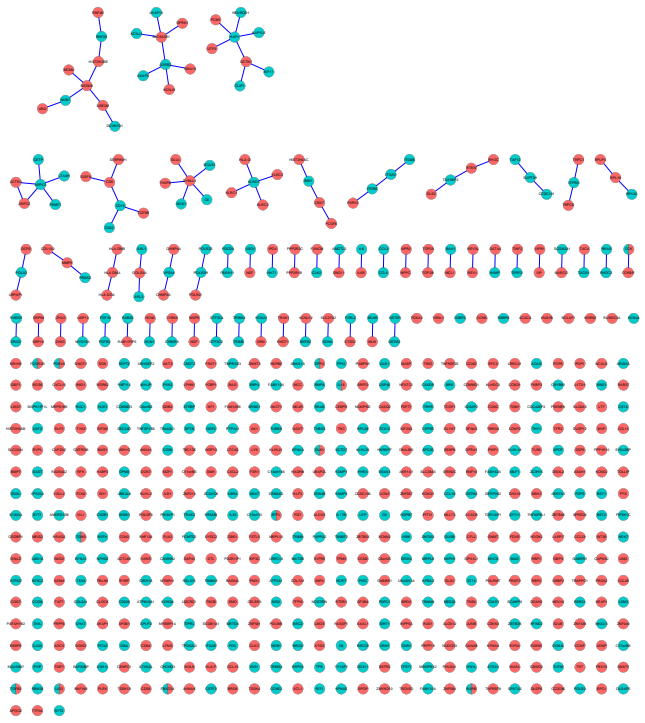

Supplement: S9 Fig — (PDF) [file pone.0123499.s009.pdf]
