# Supplementary material for: Genome-Wide Association Study of Serum Minerals Levels in Children of Different Ethnic Background
Source: PLoS One. 2015 Apr 17;10(4):e0123499. doi: 10.1371/journal.pone.0123499 (PMC4401557; doi:10.1371/journal.pone.0123499)
Supplement: S3 Fig — (PDF) [file pone.0123499.s003.pdf]

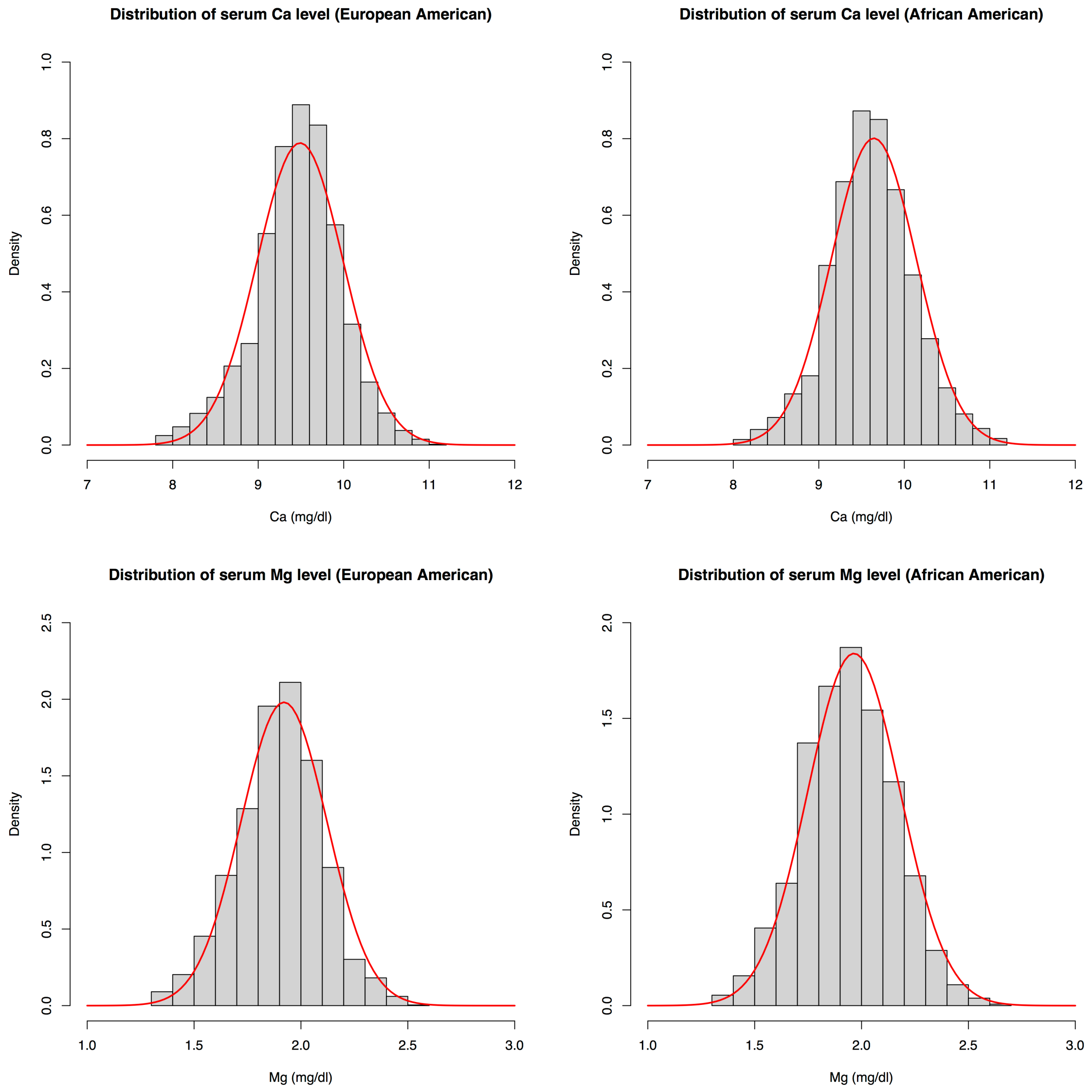
Distribution of serum Ca level (European American)
Density
Ca (mg/dl)
Distribution of serum Ca level (African American)
Density
Ca (mg/dl)
Distribution of serum Mg level (European American)
Density
Mg (mg/dl)
Distribution of serum Mg level (African American)
Density
Mg (mg/dl)
