# Supplementary material for: Genome-Wide Association Study of Serum Minerals Levels in Children of Different Ethnic Background
Source: PLoS One. 2015 Apr 17;10(4):e0123499. doi: 10.1371/journal.pone.0123499 (PMC4401557; doi:10.1371/journal.pone.0123499)

a) Association results of European-American children

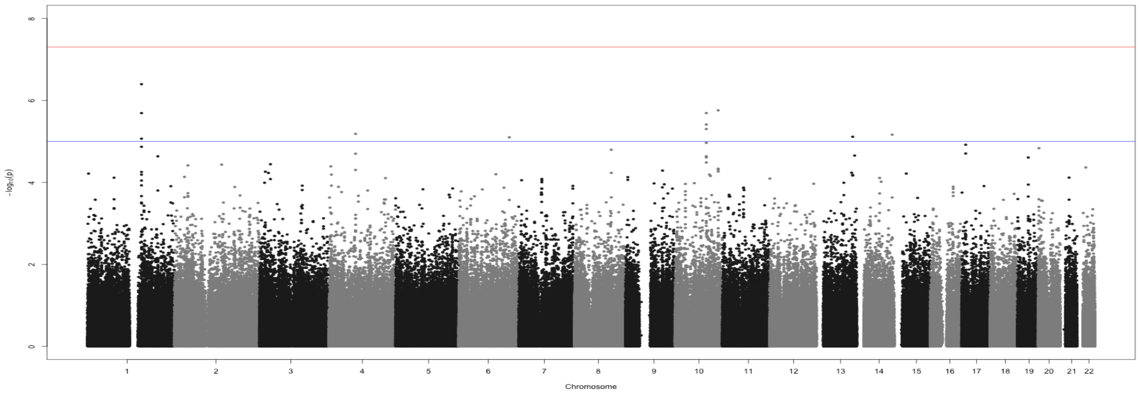

b) Association results of African-American children

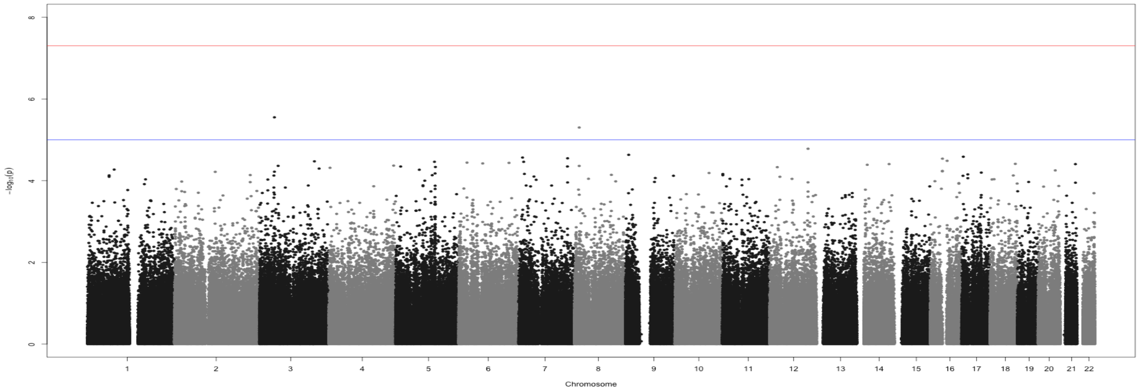

c) Meta-analysis results of European-American and African-American children

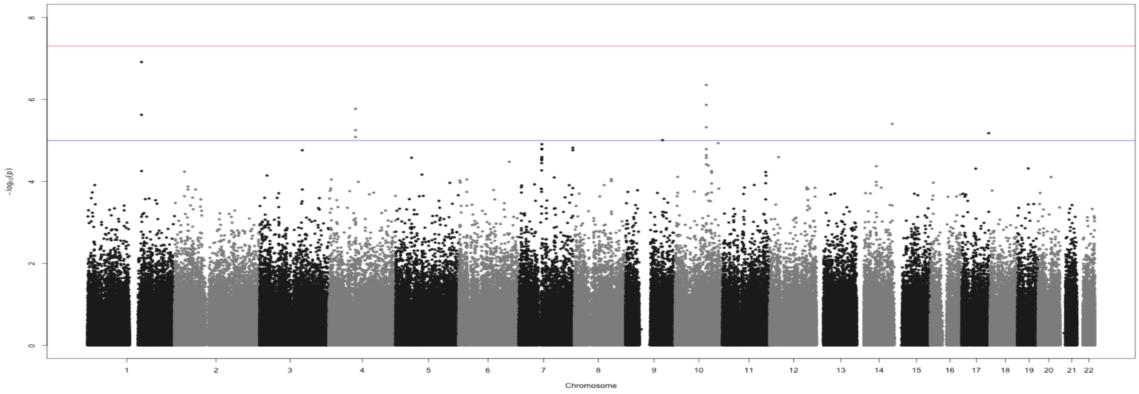

Supplement: S5 Fig — (PDF) [file pone.0123499.s005.pdf]
